# KLF7 enhances the invasion and migration of colorectal cancer cells via the miR-139-5p/TPD52 axis

**DOI:** 10.1080/15384047.2024.2385172

**Published:** 2024-08-03

**Authors:** Juan Zhang, Zhihan Li, Jiaxu Han, Zhongtao Tian, Qingyu Meng, Wenbo Niu

**Affiliations:** Department of External Medicine, The Fourth Hospital of Hebei Medical University, Shijiazhuang, China

**Keywords:** Colorectal cancer, invasion, migration, KLF7, miR-139-5p

## Abstract

In this study, we aimed to investigate the molecular mechanism of Krüppel-like factor 7 (KLF7) in colorectal cancer (CRC) cell invasion and migration. The expression pattern of KLF7 in CRC tissues and the correlation between KLF7 expression and clinical symptoms of CRC were analyzed. CRC cell lines were transfected with si-KLF7, followed by qRT-PCR or western blot detection of KLF7, miR-139-5p, and tumor protein D52 (TPD52) expression, cell counting kit-8 (CCK-8) assay to detect cell viability, and transwell detection of invasion and migration. Chromatin immunoprecipitation (ChIP) analyzed the enrichment KLF7 in the miR-139-5p promoter. The dual-luciferase reporter assay verified the binding relationship between KLF7 and miR-139-5p, and between miR-139-5p and TPD52. In the subcutaneous tumorigenesis experiment, tumor growth was observed and ki67-positive expression was detected. KLF7 is abundantly expressed in CRC cells KLF7 silencing inhibits CRC cell viability, invasion, and migration. KLF7 represses miR-139-5p expression by binding to the miR-139-5p promoter. miR-139-5p targets TPD52 expression. miR-13-5p inhibition or TPD52 overexpression partially counteracted the effect of KLF7 silencing in CRC cells. KLF7 silencing suppresses tumor growth *in vivo*. In conclusion, KLF7 suppresses miR-139-5p expression by binding to the miR-139-5p promoter, thereby upregulating TPD52 expression and enhancing CRC cell invasion and migration.

## Introduction

Colorectal cancer (CRC) is the most prevalent malignancy of the gastrointestinal tract and a paramount contributor to cancer-related deaths worldwide.^1^ The pathogenic mechanisms of CRC are relatively complicated and can be divided into three categories: chromosomal instability, microsatellite instability, and CpG island methylation phenotype.^2^ At the CRC progression stage, genetic and epigenetic changes deteriorate benign cells into malignant cells, thus further provoking invasive phenotype and metastasis.^3^ Metastatic CRC (stage IV) results in low overall 5-year survival rates, while nearly half of patients undergoing surgical treatment tend to develop metastatic CRC within 5 years, typically metastasizing to the liver, lymph nodes, and lungs.^4^ Undoubtedly, an in-depth understanding of the mechanisms underlying the invasion and metastasis of CRC is urgently needed, which can facilitate the development of novel therapeutic strategies to improve patient survival outcomes.

Krüppel-like factors (KLFs) are a group of highly conserved zinc finger transcription factors that extensively participate in a wide range of cancer-related biological processes, and aberrantly expressed KLFs function as tumor suppressors or tumor promoters in specific cellular contexts.^5^ Among the KLF family proteins, KLF7 presents an aberrant high-expression pattern in many solid tumors, including pancreatic cancer,^6^ hepatocellular carcinoma,^7^ and lung cancer.^8^ KLF7 can be employed as a powerful predictive indicator for preoperative chemoradiotherapy response in rectal adenocarcinoma patients.^9^ KLF7 mRNA expression is notably elevated in various CRC subtypes, and KLF7 overexpression is indicative of shortened disease-free survival.^10^ Nevertheless, the exact regulatory mechanism of KLF7 in CRC cell invasion and migration remains to be determined.

MicroRNAs (miRNAs) are highly conserved endogenous noncoding RNAs (∼22 nucleotides) with the capacity to control gene expression posttranscriptionally.^11^ Compelling evidence has indicated the critical role of miRNAs in CRC progression and metastasis, and dysregulated miRNAs prompt malignant phenotypes of cancer cells such as amplified proliferation and invasion, apoptosis evasion, and angiogenesis promotion.^12^ Notably, accumulating studies have revealed the aberrant downregulation of miR-139-5p in CRC samples, and miR-139-5p deficiency results in dismal prognoses and accelerates the malignant transformation of CRC cells.^13,14^ Further, miR-139-5p is reported to repress the epithelial-mesenchymal transition of CRC stem cells,^15^ strengthen the responses to chemotherapy, and depress the metastasis of CRC cells,^16^ and alleviate chronic inflammation to curb CRC cell proliferation and invasion.^17^ Hence, we speculate whether KLF7 participates in the invasion and migration processes of CRC cells by manipulating miR-139-5p expression. In the present study, we aim to determine the regulatory role of KLF7/miR-139-5p and its downstream targets in CRC cell invasion and migration, thereby conferring novel insights into the development of therapeutic targets for CRC.

## Results

### KLF7 was highly expressed in CRC and correlated with clinical symptoms of CRC

**Figure 1.**
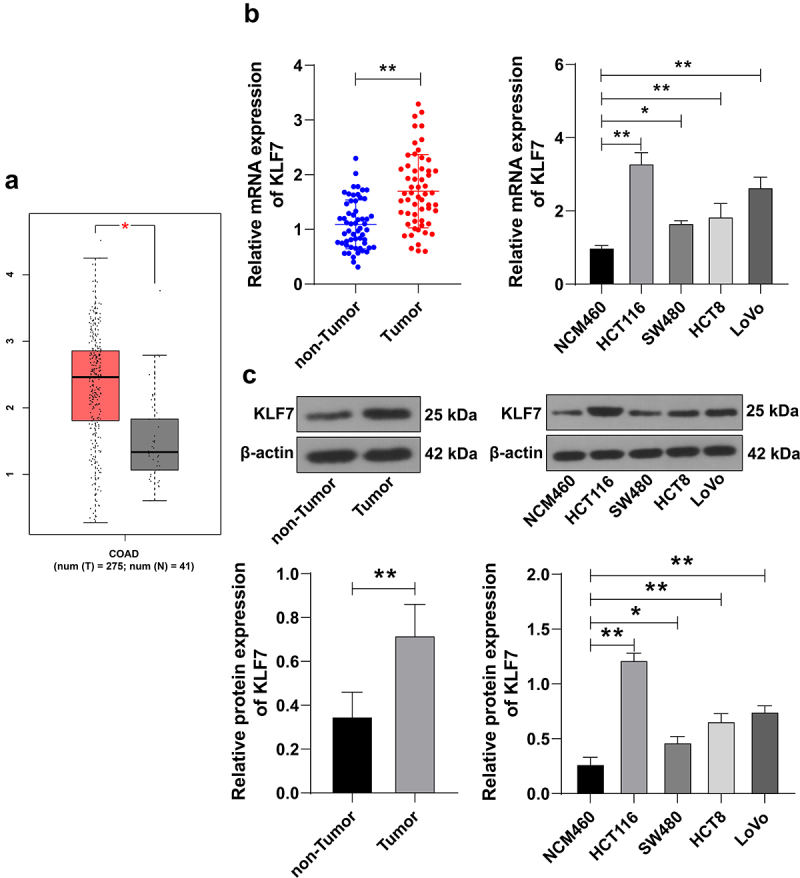
KLF7 was highly expressed in CRC. (a) GEPIA2 database online prediction showed that KLF7 was highly expressed in COAD. (b-c) KLF7 expression in cancer tissues, adjacent tissues (*n* = 57), and different cell lines was detected using qRT PCR and Western blot. The cell experiment was repeated 3 times independently. Data in panels B (left) and C (left) were analyzed using t test, and data in panels B (right) and C (right) were analyzed using one-way ANOVA, following Tukey’s multiple comparisons test, *p < .05, **p < .01.

GEPIA2 database prediction demonstrated high KLF7 expression in COAD (Figure 1(a)). We enrolled 57 CRC patients who underwent surgery in our hospital and tested KLF7 expression in cancer tissues and adjacent tissues. KLF7 showed a high expression pattern in cancer tissues (*p* < .01, Figure 1b,c). Based on the average KLF7 mRNA level, we divided the patients into the high-expression group (*n* = 25) and the low-expression group (*n* = 32). Clinical analysis indicated that KLF7 expression was significantly correlated with distant metastasis and the TNM stage of CRC (Table 1). Moreover, KLF7 expression in CRC cell lines (HCT116, SW480, HCT8, and LoVo) was notably higher than that in the normal colon epithelial cell line (NCM460) (*p* < .01, Figure 1(b,c)). HCT116 cells with relatively high KLF7 expression and SW480 cells with relatively low KLF7 expression were selected for subsequent experiments.

**Table 1.** Clinical symptom analysis of CRC patients with different KLF7 expressions.

	LOW (*n* = 32)	HIGH (*n* = 25)	*P*
Gender			0.325
Male	15	15	
Female	17	10	
Age	57.09 ± 10.38	59.40 ± 8.17	0.366
Tumor location			0.459
Colon	21	14	
Rectum	11	11	
Histological grade			
I	6	6	0.485
II	15	14	
III	11	5	
T (tumor invasion)			0.067
T1	6	1	
T2	12	5	
T3	6	11	
T4	8	8	
N (nodal status)			0.081
N0	17	6	
N1	8	11	
N2	7	8	
M (distant metastasis)			0.036
M0	24	12	
M1	8	13	
TNM stage			0.020
I	9	0	
II	4	3	
III	11	9	
IV	8	13	

Measurement data are analyzed by t test, and counting data are analyzed by Chi-squared test.

### KLF7 silencing suppressed CRC cell invasion and migration

**Figure 2.**
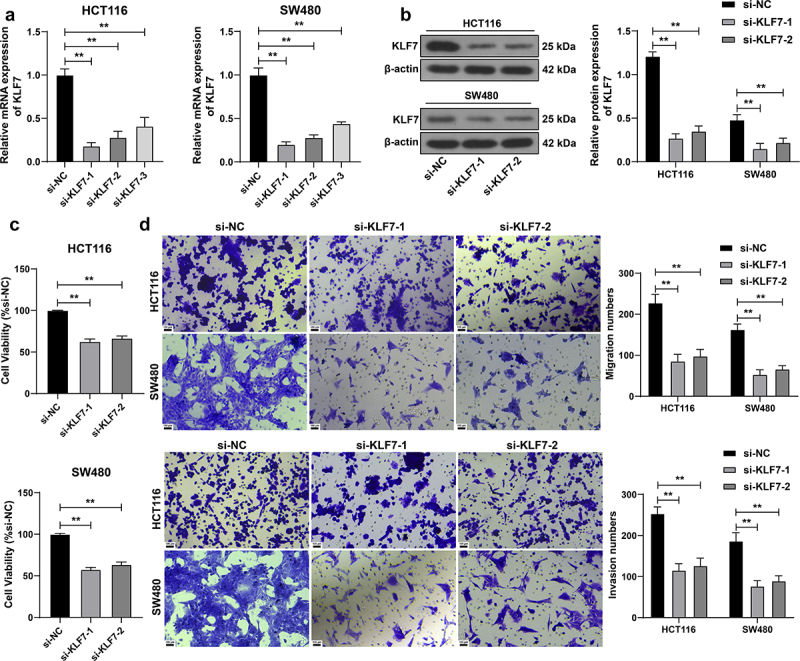
Inhibition of KLF7 reduced the invasion and migration of CRC cells. Three siRNAs targeting KLF7 (si-KLF7–1, si-KLF7–2, si-KLF7–3) were transfected into HCT116 and SW480 cells, respectively, with si-NC as the control. (a) qRT-PCR detection of transfection efficiency; si-KLF7–1 and si-KLF7–2 were selected for subsequent experiments. (b) Western blot detection of KLF7 expression. (c) CCK-8 assay detection of cell viability. (d) transwell detection of cell invasion and migration. The cell experiment was repeated 3 times independently. Data in panels AC were analyzed using one-way ANOVA, and data in panels BD were analyzed using two-way ANOVA, following Tukey’s multiple comparisons test, **p < .01.

We transfected three siRNAs targeting KLF7 into HCT116 and SW480 cells, and then selected two siRNAs (si-KLF7–1 and si-KLF7–2) with better transfection efficiency for subsequent detection (*p* < .01, Figure 2(a,b)). After silencing KLF7 expression, the viability of HCT116 and SW480 cells was dramatically diminished (*p* < .01, Figure 2(c)), and the invasion and migration abilities of cells were also reduced (*p* < .01, Figure 2(d)), indicating that KLF7 silencing curbed CRC cell invasion and migration.

### KLF7 repressed miR-139-5p transcription to upregulate TPD52 expression

**Figure 3.**
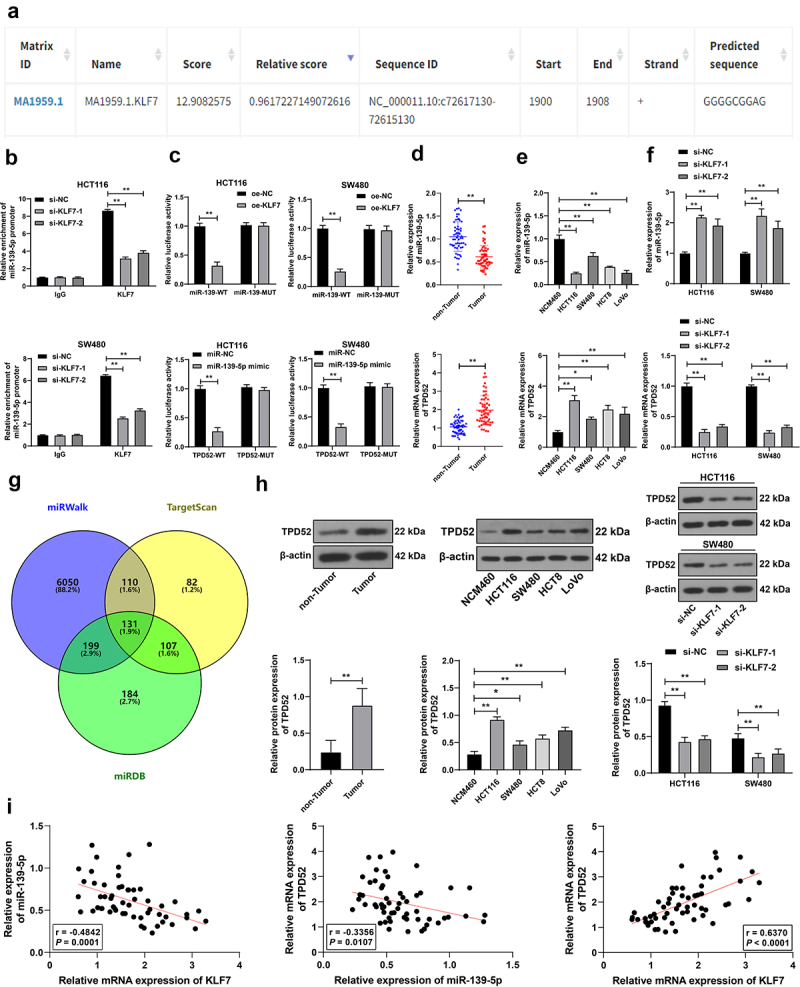
KLF7 repressed miR-139-5p transcription in CRC and thereby upregulated TPD52 expression. (a) JASPAR database prediction of the binding site of KLF7 and miR-139-5p promoter sequence. (b) ChIP analysis of KLF7 enrichment on the miR-139-5p promoter. (c) dual-luciferase reporter assay detection of the binding relationship between KLF7 and miR-139-5p promoter and the binding relationship between miR-139-5p and TPD52 3‘UTR sequence. (d-f) qRT-PCR detection of miR-139-5p and TPD52 expression in tissues (*n* = 57) and cells. (g) venn diagram of miR-139-5p downstream target genes predicted online by miRwalk, TargetScan, and miRDB databases. (h) Western blot detection of TPD52 expression in tissues (*n* = 57) and cells. I: Pearson correlation analysis of the correlation between KLF7, miR-139-5p, and TPD52 expression in tissues. The cell experiment was repeated 3 times independently. Data in panels DH (left) were analyzed using t test. Data in panels EH (middle) were analyzed using one-way ANOVA, and data in panels BCFH (right) were analyzed using two-way ANOVA, following Tukey’s multiple comparisons test, *p < .05, **p < .01.

KLF7 as a transcription factor can bind to the promoter to inhibit downstream gene expression.^18^ miR-139-5p is poorly expressed in CRC.^17^ Through JASPAR database prediction, we found that KLF7 had a binding site on the miR-139-5p promoter (Figure 3(a)). We speculated that KLF7 repressed miR-139-5p expression in CRC cells. The ChIP assay revealed that KLF7 was enriched in the miR-139-5p promoter, but the enrichment was weakened after KLF7 silencing (*p* < .01, Figure 3(b). The dual-luciferase reporter assay demonstrated that co-transfection of the KLF7 overexpression vector and miR-139-WT notably suppressed luciferase activity in the system (*p* < .01, Figure 3(c). miR-139-5p showed a low expression pattern in both CRC tissues and cells under different treatments. KLF7 inhibition resulted in increased miR-139-5p expression (*p* < .01, Figure 3(d–f)). Furthermore, we predicted the downstream targets of miR-139-5p using the miRWalk, TargetScan, and miRDB databases (Figure 3(g)), among which we focused on TPD52. TPD52 expression is reported to be elevated in CRC.^19,20^ The dual-luciferase reporter assay confirmed that the binding of miR-139-5p to the TPD52 3‘UTR sequence notably repressed luciferase activity (*p* < .01, Figure 3(c)). TPD52 was highly expressed in CRC tissues and cells (*p* < .01, Figure 3(d–f,h). Pearson correlation analysis revealed a negative correlation between KLF7 and miR-139-5p and a positive correlation between KLF7 and TPD52 (*p* < .01, Figure 3(i). These results comprehensively indicated that KLF7 repressed miR-139-5p transcription in CRC, thereby upregulating TPD52 expression.

### miR-139-5p silencing partially counteracted the effect of KLF7 silencing on CRC cell invasion and migration

**Figure 4.**
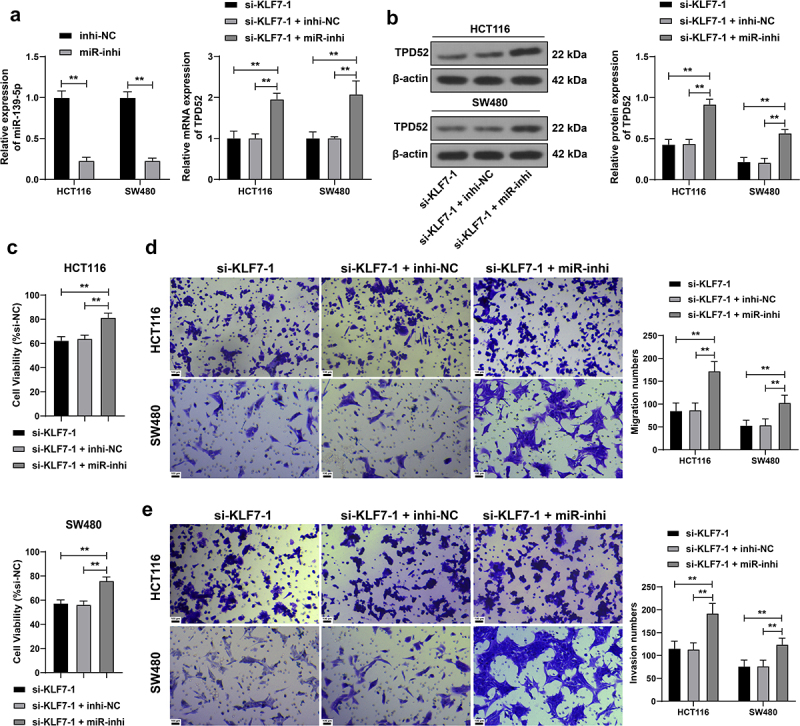
Inhibition of miR-139-5p partially reversed the effect of KLF7 silencing on CRC cell invasion and migration. miR-139-5p inhibitor (miR-inhi) was transfected into HCT116 and SW480 cells, with inhi-NC as the control, followed by a combined experiment with si-KLF7–1. (a) qRT-PCR detection of miR-139-5p and TPD52 expression. (b) Western blot detection of TPD52 expression. C: CCK-8 assay detection of cell viability. (d-e) transwell detection of cell invasion and migration. The cell experiment was repeated 3 times independently. Data in panel C were analyzed using one-way ANOVA, and data in panels ABDE were analyzed using two-way ANOVA, following Tukey’s multiple comparisons test, **p < .01.

We downregulated miR-139-5p expression in HCT116 and SW480 cells (*p* < .01, Figure 4(a) for a combined experiment with si-KLF7–1 to verify the above mechanism. Compared with KLF7 silencing alone, the combined treatment elevated TPD52 expression (*p* < .01, Figure 4(b), enhanced cell viability (*p* < .01, Figure 4(c), and strengthened invasion and migration (*p* < .01, Figure 4(d–e), indicating that inhibiting miR-139-5p partially reversed the inhibitory effect of KLF7 silencing on CRC cells.

### TPD52 overexpression partially offset the effect of KLF7 silencing CRC cell invasion and migration

**Figure 5.**
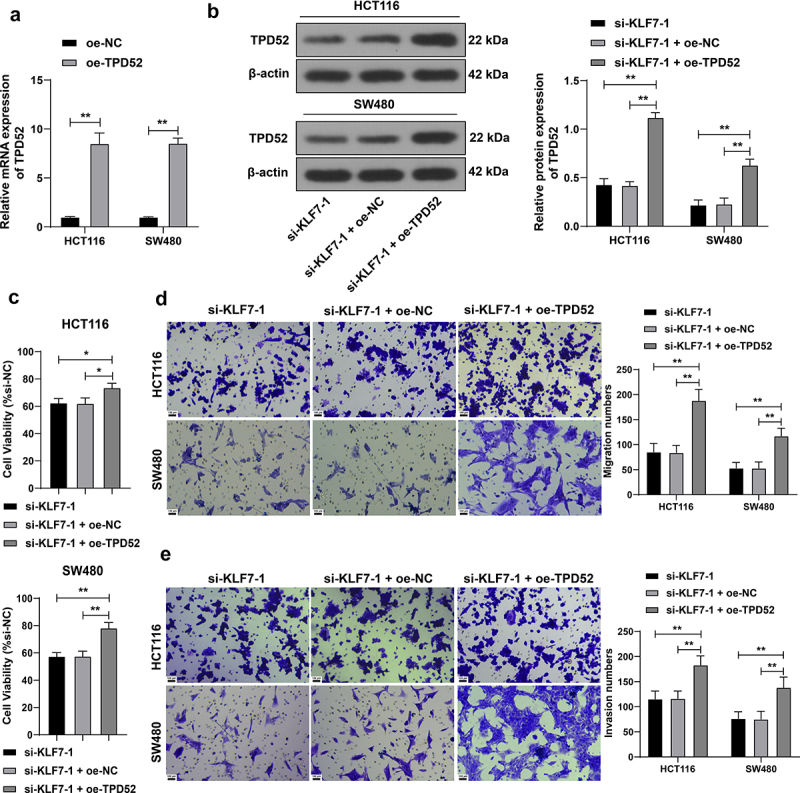
Overexpression of TPD52 partially reversed the effect of KLF7 silencing CRC cell invasion and migration. TPD52 overexpression vector (oe-TPD52) was transfected into HCT116 and SW480 cells, with empty vector (oe-NC) as the control, followed by a combined experiment with si-KLF7–1. (a) qRT-PCR detection of TPD52 expression. (b) Western blot detection of TPD52 expression. (c) CCK-8 assay detection of cell viability. (d-e) transwell detection of cell invasion and migration. The cell experiment was repeated 3 times independently. Data in panel C were analyzed using one-way ANOVA, and data in panels ABDE were analyzed using two-way ANOVA, following Tukey’s multiple comparisons test, *p < .05, **p < .01.

We overexpressed TPD52 in HCT116 and SW480 cells (*p* < .01, Figure 5(a,b) for a combined experiment with si-KLF7–1. Compared with KLF7 silencing alone, the combined treatment resulted in augmented CRC cell viability (*p* < .01, Figure 5(c) and enhanced invasion and migration (*p* < .01, Figure 5(d–e), indicating that TPD52 overexpression partially counteracted the inhibitory effect of KLF7 silencing on CRC cells.

### KLF7 silencing repressed tumor growth in mice

**Figure 6.**
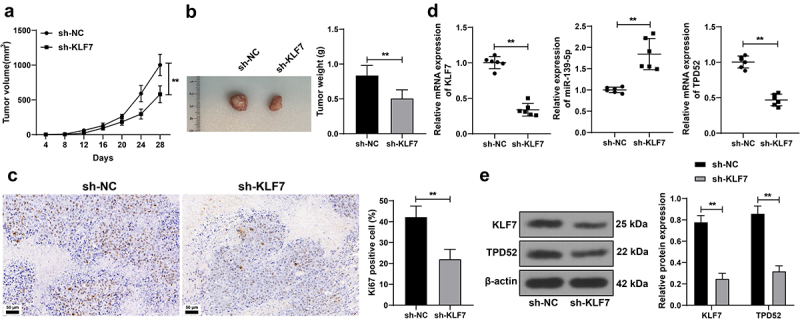
Inhibition of repressed tumor growth in mice. Mice were subcutaneously injected with HCT116 cells stably low-expressing KLF7 (sh-KLF7), with sh-NC as the control. (a) tumor volume measured with vernier scale every 4 days, *n* = 12. (b) tumor weight and representative tumor images, *n* = 12. (c) IHC detection the ki67-positive expression in the tumor, *n* = 6. (d) qRT-PCR detection of KLF7, miR-139-5p, and TPD52 expression in the tumor. (e) Western blot detection of KLF7 and TPD52 expression in the tumor, *n* = 6. Data in panels BCD were analyzed using t test, and data in panels AE were analyzed using two-way ANOVA, following Tukey’s multiple comparisons test, **p < .01.

Finally, we validated the effect of KLF7 on tumor growth in mice. KLF7 inhibition restricted tumor growth (*p* < .01, Figure 6(a,b) and declined the ki67-positive rate in the tumor (*p* < .01, Figure 6(c). After KLF7 silencing, miR-139-5p expression was elevated, whereas TPD52 expression was reduced (*p* < .01, Figure 6(d–e). These results indicated that KLF7 silencing restrained CRC tumor growth *in vivo*.

## Discussion

**Figure 7.**
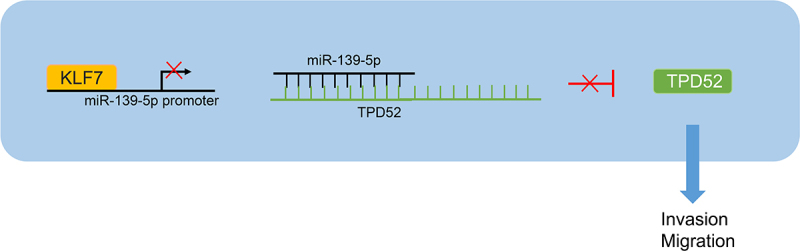
KLF7 is highly expressed in CRC cells and inhibits miR-139-5p expression by binding to the miR-139-5p promoter, thereby reducing the targeted inhibition of miR-139-5p on TPD52 and increasing TPD52 expression, ultimately enhancing the invasion and migration of CRC cells.

CRC is a complex heterogeneous disease in which individuals may exhibit accumulation of somatic mutations at different stages, leading to malignant and invasive phenotypes in cells.^3^ A plenty of studies have pointed out the detrimental effects of KLF7 on carcinogenesis and cancer progression, including endometrial cancer,^21^ hepatocellular carcinoma,^7^ and CRC.^10^ We are the first to discover the mechanism of KLF7/miR-139-5p/TPD52 in CRC cell invasion and migration (Figure 7).

The KLF family of proteins is essential for the normal functioning of diverse biological processes. KLF proteins frequently exhibit dysregulated expression profiles in CRC and possess prominent prognostic values.^5^ A significant association between KLF7 mRNA and CRC recurrence has been observed in a published microarray dataset.^22^ The aberrant KLF7 overexpression magnifies the clonogenic activity of CRC cells and predicts dismal prognoses in CRC patients.^10^ Our data were consistent with the previously published findings that KLF7 was abundantly expressed in CRC and KLF7 silencing attenuated CRC cell invasion/migration and also restrained tumor growth *in vivo*.

Currently, mechanistic insights into the role of KLF7 in CRC progression are limited. Therefore, this study aimed to explore the downstream mechanism of KLF7 mediating CRC cell invasion and migration. The interactions between transcription factors and miRNAs affect gene regulatory networks, thereby manipulating the pathogenesis and developmental progression of various cancers.^23^ KLF7 as a transcription factor was predicted to have a binding site with miR-139-5p. ChIP and dual-luciferase reporter assays further verified the binding between KLF7 and miR-139-5p. miR-139-5p is deemed an available tumor suppressor in CRC, as it assists in suppressing CRC cell proliferation in vitro and curbing tumorigenicity and metastasis *in vivo*.^16,24,25^ miR-139-5p weakens the stemness maintenance and depresses the epithelial-mesenchymal transition of CRC stem-like cells, implying the potential of miR-139-5p in the clinical treatment of recurrent and metastatic CRC.^15^ We found that miR-139-5p was notably diminished in CRC tissues and cells, and KLF7 silencing resulted in an increase in miR-139-5p expression. Functional rescue experiments verified that miR-139-5p inhibition partially offsets the inhibitory effect of KLF7 silencing on CRC cell invasion and migration.

Furthermore, we predicted the downstream targets of miR-139-5p using online databases, among which we focused on TPD52. TPD52, a member of the similarly named gene and protein family, shows an overexpression pattern and increased copy number in multiple human malignancies.^26,27^ Consistently, we observed an upregulation of TPD52 in CRC tissues and cells. Pearson correlation analysis revealed a negative correlation between KLF7 and miR-139-5p and a positive correlation between KLF7 and TPD52, indicating that KLF7 repressed miR-139-5p transcription in CRC by upregulating TPD52 expression. TPD52 was found to be dramatically elevated in CRC samples, independent of tumor localization, grading, and TNM staging.^20^ TPD52 overexpression induces the migration/invasion and epithelial-mesenchymal transition of CRC cells and serves as an independent predictor for reduced overall survival.^19^ We also observed that TPD52 overexpression enhanced the viability, invasion, and migration of CRC cells treated with KLF7 silencing, suggesting that TPD52 overexpression counteracted the inhibitory effect of KLF7 silencing on CRC cell invasion and migration.

In conclusion, KLF7 overexpression in CRC cells suppressed miR-139-5p expression and attenuated the targeted inhibition of miR-139-5p on TPD52, thereby upregulating TPD52 expression and eventually augmenting CRC cell invasion and migration. However, this study had certain limitations. Our animal experiments merely observed tumor growth but did not verify invasion and migration. Moreover, the sample size was relatively small. The downstream target genes of KLF7 and miR-139-5p are all single selections, and the roles of other target genes in CRC cells remain to be determined. At present, the experimental conditions and funds of our research group are insufficient to support the relevant sequencing and microarray analysis, so we cannot evaluate all target genes downstream of KLF7. In the future, we will apply ChIP-seq or microarray analysis to analyze all target genes downstream of KLF7 and screen key genes through differential expression analysis, so as to establish the regulatory network of KLF7 in CRC. In future research, we will further verify the KLF7/miR-139-5p/TPD52 axis in other physiological and biochemical processes of CRC cells and determine the roles of more target genes in CRC cellular processes, thereby conferring novel insights into the interventions of CRC progression and metastasis.

## Material and methods

### Ethics statement

This study was approved by the Ethics Committee of the Fourth Hospital of Hebei Medical University. All procedures involving human participants were carried out in accordance with the *Declaration of Helsinki*, and written informed consent was obtained from all participants. All the animal experimental protocols were approved by the Animal Ethics Committee of the Fourth Hospital of Hebei Medical University.

### Clinical sample

A total of 57 patients with CRC who underwent surgery at our hospital between January 2020 and January 2022 were recruited. Pathological symptoms were confirmed through endoscopic biopsy and imaging before surgery. The cancerous and adjacent tissues were surgically removed and quickly transferred to liquid nitrogen for preservation. Inclusion criteria: 1. Aged 20–80 years old; 2. Karnofsky scores ≥ 80,^28^ 3. No obvious lesions (including malignant lesions, polyps, and ulcers) were found during gastroscopy within 3 months; 4. Written informed consent was obtained from patients and their families. Exclusion criteria:1. American Society of Anesthesiologists (ASA) grade IV-V;^29^ 2. Pregnant and lactating women; 3. Serious cardiovascular disease, uncontrollable infection, or other uncontrollable concomitant diseases; and 4. History of multiple tumors or other genetic disorders. The clinicopathological features recorded for this study included tumor size and location, histological grading, and tumor-node-metastasis (TNM) staging. TNM staging included tumor invasion (T), regional lymph node status (N), and metastasis (M).

### Cell culture

Human CRC cell lines (HCT116, SW480, HCT8, and LoVo) and a normal human colon epithelial cell line (NCM460) purchased from ATCC (Manassas, Virginia, USA) were incubated in Dulbecco’s modified Eagle’s medium (DMEM) supplemented with 100 U/mL penicillin-streptomycin and 10% fetal bovine serum (FBS) in a humidified incubator containing 5% CO_2_ and 95% air at 37°C.

### Cell treatment

Three siRNAs targeting KLF7 (si-KLF7–1, si-KLF7–2, and si-KLF7–3) and their corresponding si-NC were designed. The tumor protein D52 (TPD52) overexpression vector pcDNA3.1-TPD52 (oe-TPD52) was constructed with an empty vector (oe-NC) as the control. The miR-139-5p inhibitor (miR-inhi) and inhibitor control (inhi-NC), as well as the above vectors and sequences, were designed and synthesized by GenePharma (Shanghai, China). The above vectors and sequences were transfected into cells using Lipofectamine 3000 (Invitrogen, Carlsbad, CA, USA; Thermo Fisher Scientific, Waltham, MA, USA).

### Cell counting kit-8 (CCK-8) assay

Cell viability was evaluated using a CCK-8 assay kit (Sangon Biotechnology Co., Ltd., Shanghai, China). The cells were seeded into 96-well plates (2 × 10^3^ cells/well) and cultured for 48 h. Then, 10 µL of the CCK-8 solution was added to each well and incubated at 37°C for 4 h. Absorbance was measured at 450 nm using a microplate reader (Thermo Fisher Scientific). Each group was set up in three wells, and the experiment was independently repeated three times.

### Transwell

Cell invasion was detected using a Matrigel invasion assay (BD Biosciences, San Jose, CA, USA). Briefly, the cells were collected and suspended in serum-free medium. Cells (1 × 10^5^) were diluted with 500 µL serum-free medium and incubated in the apical chamber of the Transwell device (Corning Glass Works, Corning, NY, USA) at 37°C for 2 h, and the apical chamber was pre-coated with 1 mg/mL matrix (BD Biosciences). Subsequently, a culture medium containing 10% FBS was added to the basolateral chamber. After incubation for 48 h, the cells invading the Matrigel membrane were fixed with 4% paraformaldehyde at room temperature for 30 min, stained with 0.5% crystal violet for 30 min, and counted in five high-power fields (magnification × 200). Three independent experiments were conducted. For the migration experiment, except for the absence of Matrigel in the apical chamber, the remaining experimental procedures were the same as those in the invasion experiment.

### Quantitative real-time polymerase chain reaction (qRT-PCR)

Total RNA was extracted using TRIzol reagent (Invitrogen) and quantified using a NanoDrop2000 instrument (Thermo Fisher Scientific). The TaqMan MicroRNA kit (Applied Biosystems, Carlsbad, CA, USA) was used for miRNA detection. miRNA expression was standardized with the internal reference U6.^30^ PrimerScript™RT Reagent kit (Takara, Tokyo, Japan) was used for cDNA synthesis, and qPCR was performed using the FastStart Universal SYBR Green Master kit (Roche Diagnostics, Indianapolis, Indiana, USA). mRNA expression was standardized to GAPDH mRNA expression. The primer sequences used are listed in Table 2. The relative gene expression was calculated using the 2^−ΔΔCt^ method.^31^

**Table 2.** PCR primer sequences.

Name	Sequence (5’-3’)
KLF7	F: AGACATGCCTTGAATTGGAACG
	R: GGGGTCTAAGCGACGGAAG
miR-139-5p	F: GCCGAGTCTACAGTGCACGTG
	R: CTCAACTGGTGTCGTGGA
TPD52	F: AGCATCTAGCAGAGATCAAGCG
	R: AGCCAACAGACGAAAAAGCAG
GAPDH	F: GGAGCGAGATCCCTCCAAAAT
	R: GGCTGTTGTCATACTTCTCATGG
U6	F: TCGCTTCGGCAGCACATATACT
	R: AGGTGGCTTTGGTGGAAGAG
miR-139-5p promoter	F: CATCCACCCCCAAACCTAGCTCCTG
	R: GAGCCAGTCCCAGTGCCTCCCAAGG

KLF7, KLF transcription factor 7; miR-139-5p, microRNA-139-5p; TPD52, tumor protein D52; GAPDH, glyceraldehyde-3-phosphate dehydrogenase.

### Western blot

Total protein was extracted using radioimmunoprecipitation buffer (Beyotime, Shanghai, China) and protease inhibitor cocktail (Roche Diagnostics). Protein concentration was measured using a bicinchoninic acid assay kit (Thermo Fisher Scientific), and 30 µg of total protein was separated by 10% SDS-PAGE and transferred onto PVDF membranes (EMD Millipore, Billerica, MA, USA). Membranes were incubated with 5% skim milk for 1 h to block nonspecific binding, followed by incubation with primary antibodies against KLF7 (1:2000, NBP2–99217, NOVUS, Littleton, CO, USA), TPD52 (1:1000, ab234819, Abcam, Cambridge, MA, USA), and β-actin (1:1000, ab8227, Abcam) at 4°C overnight. After washing, the membranes were incubated with horseradish peroxidase-conjugated IgG (1:1000, ab6721, Abcam) for 30 min and analyzed using an automatic chemiluminescence image analysis system (Tanon 6100, Shanghai Tanon Technology Co., Ltd., Shanghai, China).

### Bioinformatics

The expression of KLF7 in COAD (colon adenocarcinoma) was searched through the GEPIA2 database (http://gepia2.cancer-pku.cn/#index).^32^ The binding site between KLF7 and miR-139-5p promoter was analyzed through the JASPAR database (https://jaspar.genereg.net/).^33^ The downstream target genes of miR-139-5p were predicted through the miRWalk database (http://mirwalk.umm.uni-heidelberg.de/),^34^ TargetScan database (http://www.targetscan.org/),^35^ and miRDB database (http://mirdb.org/).^36^

### Chromatin immunoprecipitation (ChIP)

The EZ-Magna ChIP A/G kit (Millipore) was used for ChIP detection of KLF7 enrichment in the miR-139-5p promoter. The cells were cross-linked with 1% formaldehyde, quenched with glycine, and subjected to ultrasound treatment. The supernatant was mixed with Magna ChIP Protein A/G Magnetic Beads (preincubated with KLF7 (1:2000, NBP2–99217, NOVUS) or IgG (1:2000, ab171870, Abcam) antibodies) at 4°C. The immunoprecipitated complex was collected, eluted, and incubated with proteinase K at 65°C for 2 h and at 95°C for 15 min. The protein was removed by reverse crosslinking. The eluted DNA was used as a template for qPCR analysis, and the primers used are listed in Table 2.

### Dual-luciferase reporter assay

The miR-139-5p promoter sequence containing the KLF7 binding site and the mutant sequence was cloned into a dual-luciferase reporter vector (Promega, Madison, WI, USA) to construct the miR-139-WT and miR-139-MUT reporting vectors. Meanwhile, the TPD52 3‘UTR fragment covering the miR-139-5p binding site and the mutation sequence on the target site were amplified and inserted into the pmirGLO reporter luciferase vector (Promega) to construct TPD52-WT or TPD52-MUT. The cells were then seeded into 24-well plates and co-transfected with the constructed luciferase vectors, KLF7 overexpression vector (oe-KLF7), miR-139-5p mimic or miR-NC, and empty vector (oe-NC). After 48 h, the Dual-luciferase® Reporter Gene Analysis System (Promega) was used to detect the luciferase activity.

### Tumorigenesis in nude mice

Female BALB/c nude mice (4–6 weeks old, weighing 20–30 g; Guangdong Medical Experimental Animal Center, Foshan, Guangdong, China) were kept in a sterile room at 24 ± 2°C, 60–80% humidity, and a 12-h light/dark cycle, with free access to food and water. HCT116 cells stably expressing KLF7 (infected with lentivirus-packaged sh-KLF7 and screened by puromycin) were injected subcutaneously into the left side of the mice (5 × 10^6^ cells/mouse). The tumor size was measured using a Vernier scale every 4 days, and the tumor volume was calculated as follows: volume = (length × width × width)/2. On the 28th day, the animals were euthanized and the tumor was removed and quickly frozen. Tumor tissues were randomly selected from six mice in each group for formaldehyde fixation and paraffin embedding, while the tissues of the remaining six mice were homogenized for detection.

### Immunohistochemistry

After dewaxing and hydration, tumor sections were incubated with anti-ki67 (1:200, ab16667, Abcam) at 4°C and then reacted with IgG (1:2000, ab205718, Abcam) at 37°C for 2 h. After washing with phosphate-buffered saline, the color development reaction was performed with 2,4-diaminobutyric acid as the substrate and H_2_O_2_ as the catalyst. After sealing, the sections were observed and analyzed under a microscope.

### Statistical analysis

Data analysis and map plotting were performed using SPSS 21.0 (IBM Corp., Armonk, NY, USA) and GraphPad Prism 8.0 (GraphPad Software Inc., San Diego, CA, USA). The data were examined for normality and homogeneity of variance. Counting data were analyzed using the chi-squared test or Pearson correlation analysis. For measurement data, the *t* test was used for comparisons between two groups, and one-way or two-way analysis of variance (ANOVA) was used for comparisons among multiple groups, following Tukey’s multiple comparison test. A value of *p* < .05 indicated a significant difference.
